# Health care priority setting in Norway a multicriteria decision analysis

**DOI:** 10.1186/1472-6963-12-39

**Published:** 2012-02-15

**Authors:** Thierry Defechereux, Francesco Paolucci, Andrew Mirelman, Sitaporn Youngkong, Grete Botten, Terje P Hagen, Louis W Niessen

**Affiliations:** 1 Department of International Health, Johns Hopkins School of Public Health, 615 N Wolfe Street, Baltimore, USA; 2 Department of Health Management and Health Economics, University of Oslo, PO Box 1089, Blindern, NO-0317 Oslo, Norway; 3 Australian Centre for Economic Research on Health, College of Medicine, Biology and Environment, The Australian National University, M Block, cnr Mills & Eggleston Roads, Canberra ACT 0200, Australia; 4 Health Intervention and Technology Assessment Program, Department of Health, Ministry of Public Health, Nonthaburi 11000, Thailand

## Abstract

**Background:**

Priority setting in population health is increasingly based on explicitly formulated values. The Patients Rights Act of the Norwegian tax-based health service guaranties all citizens health care in case of a severe illness, a proven health benefit, and proportionality between need and treatment. This study compares the values of the country's health policy makers with these three official principles.

**Methods:**

In total 34 policy makers participated in a discrete choice experiment, weighting the relative value of six policy criteria. We used multi-variate logistic regression with selection as dependent valuable to derive odds ratios for each criterion. Next, we constructed a composite league table - based on the sum score for the probability of selection - to rank potential interventions in five major disease areas.

**Results:**

The group considered cost effectiveness, large individual benefits and severity of disease as the most important criteria in decision making. Priority interventions are those related to cardiovascular diseases and respiratory diseases. Less attractive interventions rank those related to mental health.

**Conclusions:**

Norwegian policy makers' values are in agreement with principles formulated in national health laws. Multi-criteria decision approaches may provide a tool to support explicit allocation decisions.

## Background

In all health care systems, choices in the allocation of resources are necessary. Public resources, in both low- and high-income settings, are insufficient to provide all possible services to the entire population at all times. Priority setting in the allocation of health interventions across ranges of health services or target groups becomes inevitably and at best should be explicit[1,2].

Scarcity of resources and rational priority setting lead to identification of national packages of health services and to explicit reimbursement decisions [3]. In health, policy makers often may have to confront many groups with different and conflicting interests [4]. Rational approaches to guide policy makers become desirable and allow for navigation of complex social processes and to handle multifaceted information. The rise of evidence-based health policy has lead to a wealth of information on the nature and distribution of the disease burden and related societal costs as well on the effectiveness and efficiency of health interventions, and on their budget impact [5]. Existing approaches may concentrate on one or two criteria like safety and effectiveness, or also efficiency [6,7] with real world decision makers having to handle multidimensional information [2,8]. So far, priority setting tends to focus primarily on effectiveness and efficiency and less on equity-related social values [9].

Norway has adopted a well-developed comprehensive system in which complex problems and scientific support mechanisms such as health technology assessment and value-based priority-setting criteria were brought together to generate transparent solution [10]. As one of the richest countries it ranks second following the United States in health care spending and it enjoys one of the highest population health levels in the world. (OECD 2011[11]) The key elements of its health policy include provisions of health services for all based on needs regardless of personal income, local and regional accountability, public commitment and political interest in improving the health system [12]. Its health system is described as a decentralized national health service with universal coverage of primary care under the responsibility of the municipalities and with specialized care under national governance.

In 2007 a Council for Quality Improvement and Priority Setting was established *"to debate, implement, regulate, and supervise" *the national health plan under the Patient Rights Act of 2001. As such, the Norway situation provides a unique opportunity to compare formal health goals to actual policy makers preferences in priority setting.

Our study objective is to assess if policy-makers' actual value preferences agree with those formulated in the legal domain through the Patient Rights Act (PRA). The Act identifies three prioritizing rules: severity of the condition (those most in need must be helped first), expected health outcome, and, lastly, proportionality between need and treatment (with cost-effectiveness analysis providing this information). We used discrete choice experiments (DCE), a methodology that allows simultaneous assessment of multiple policy preferences in multi-criteria decision analysis (MCDA) for policy as we reported earlier in other case studies[9,13-15].

## Methods

MCDA consists of four steps: (I) identification of policy criteria and metrics, (II) identifying series of alternative package vignettes based on various combinations of policy criteria, (III) measuring performance of alternatives by criteria (this is often done through a DCE questionnaire but can also be completed in various other ways), and (IV) determining the preferred selection through scoring intervention options against those criteria. The last step provides a broad classification and ranking of interventions within a chosen context or specific disease area (a composite league table). Although the league table is not an integral part of the MCDA methods but rather an illustrative application, it is often seen as the most important contribution of a MCDA.

### Discrete choice survey and criteria attributes

A DCE is a stated-preference technique that uses an attribute-based survey method for measuring preferences for multiple benefits (utility). The survey elicits respondents' preferences based on their stated preferences in hypothetical and forced choices between two vignettes or, in our study, two sets of specified policy criteria. The choice vignettes are drawn, *a priori*, from all possible choice sets according to statistical design principles [16]. In stating a preference for one of the two vignettes, the individual is assumed to make trade-offs and choose the alternatives that yield the highest personal preference or benefit or utility [17].

The first step is the identification of context-relevant criteria and their levels. In general, criteria must be relevant to the stated policy choices and usually include some combination of equity-related and/or efficiency-related factors. Here, equity factors may be viewed as related to the distributional impact and often relate to whom in a more vulnerable state receives the expected health benefits like those with severe illness, of a high or low age or low income status. Efficiency factors may refer to achieving the largest impact at the lowest cost, the total beneficiaries and hence the total population impact, or the individual benefits. We used an existing questionnaire, applied in various settings and reported earlier for other countries, including The Netherlands [14,15,18]. The criteria (Table 1) are selected to assure completeness, feasibility, and mutual independence [3,9], as based in the earlier country experiences and review [6,7].

**Table 1:** Definition of attributes and levels

Attribute	Level	Definition
Severity of disease	Not severe	Health expectancy > 2 years without intervention
	Severe	Health expectancy < 2 years

Number of potential beneficiaries	Few	< 100 000
	Many	> 100 000

Age of target group	Young age	0-15 years old
	Middle-age	15-59 years old
	Elderly	> 60 years old

Individual health benefits	Small	< 5 healthy years
	Large	> 5 healthy years

Willingness to subsidize	> 70% of total health expenditure	Poverty reduction criteria: subsidize at more or less than 70%.
	< 70% of total Health expenditure	

Cost-effectiveness	Not C-E	Cost/DALY > GDP/capita
	C-E	Cost/DALY< GDP/capita

*DALY *Disability-adjusted life year

*GDP *Gross domestic product

The survey questionnaire is detailed in Table 2 with the varying levels of criteria attributes that are grouped and assigned into sets of vignettes. Vignettes contain always the same attributes yet with differing levels across vignettes. A full factorial list, including all possible combination of levels and attribute is often impossible to use, yet not needed. We applied a fractional factorial design considering a sample selection of the possible alternatives. A computer-based software program was used to create choice sets regarding consideration on the properties of orthogonality, level balance, and minimum overlap [2]. Our fractional factorial design in the study includes a sub-set of 32 vignettes, paired in 16 sets of choices.

**Table 2:** Pair of competing interventions example in the DCE questionnaire scenarios.

Choice	A	B
Severity of disease	Severe	Not severe
Number of potential beneficiaries	Many	Few
Age of target group	Mid age	Old age
Individual health benefits	Large	Small
Willingness to subsidize	> 70%	< 70%
Cost-effectiveness	Cost effective	Not Cost-effective
YOUR CHOICE: tick a box	A□	B□

If these were your only options, which would you choose, tick a box: A or B

The DCE consists of this set of discrete choices. It measures preferences between intervention options by counting the extent to which specified objectives are preferred most [2,3]. We used conditional logistic regression model in STATA ^© ^to analyze all the response data together. The independent variables (the defined policy criteria) are either categorical with two or three levels and the dependent variable is the preferred choice i.e. the selected vignette. In case of a three levels attribute, the young age group is used as the reference category. The weighing results are presented as regression coefficients. These indicate the size of the effect of a criterion on the selection probability of an intervention option fulfilling the criterion [16]. Finally, the calculated sum weight over all criteria for each intervention option leads to ranking of the options in a composite league table based on the probability of selection [14].

### Data collection

The DCE survey questionnaires were completed by 34 health professionals selected among all the senior members of the Norwegian Directorate of Health and senior public health academic officials, using a snow-balling technique. The selection of respondents was based on the number of years with relevant experience in actual decision-making, or their familiarity with economic evaluation defined as being actively involved in policy research in this field.

### Data analysis

All levels for criteria were qualitative and data are dichotomous choice ('1' represents the option being chosen, '0' where not chosen). The observed sources of utility can be defined as a linear expression (main-effects additive probability model assumption) in which each attribute is weighted by a unique parameter to account for that attribute's marginal utility. Regression coefficients indicate the sign of the effect of a variable on the probability of selection of an intervention. The absolute value of the coefficients denote the relative importance of particular levels of a criterion in comparison to other levels of all other criteria so that the criterion which has higher probability will have more possibility to be used as the important one in selection of the interventions than others [16,17].

### Composite league table

The last phase in MCDA includes the ranking of interventions of interest in the country where the research is performed. In an actual priority-setting context, the aim of the composite league table is to rank-order the interventions to identify those that should be included or excluded from a health initiative (low-income countries) or public reimbursement (high-income settings). We selected the interventions in the Norway context to provide a broad presentation of existing and possible interventions across several disease areas, to demonstrate how MCDA may support rational decisions [7].

In the five major disease burden groups (WHO, 2011[19]) we selected 21 interventions. We consulted the updated National Clinical Guidelines as issued by the Directorate for Health and other sources to find the most appropriate context-given interventions addressing the five disease groups. The 21 interventions addressed neuropsychiatric conditions, cardio-vascular diseases, malignant neoplasms, respiratory diseases, and sensory organ diseases.

We defined a "composite index" (CI) that represents the relative priority of each intervention as a function of their characteristics, based on the criteria weights. The "probability of selection" is estimated for each intervention using the regression model and a rank ordering of all intervention on the basis of this composite index results in a composite league table [15].

Our main effect model uses the following specification:

(1) $$Logit\;\left(P\right)=Ln\;\left(P/1-P\right)=\beta 0+\beta_{i}V_{i}+\beta_{i}V_{i}+\ldots +\varepsilon \;\text{(1)}$$

*P *is the probability of an intervention to be selected as the most qualifying intervention based on the joint inclusion of all relevant criteria and their relative weights. The *β0 *is the constant term or intercept, the *βi + n *are the indexed coefficients for each the criteria included in the model, while ε is the unobservable error term. *V *has the value of '1' in case the criterion is present and '0' when it is absent. The use of this linear additive utility model is based on the assumption of mutual independence of the criteria selected.

Computation of the CI involves several steps. The set of interventions to be prioritized has to be mapped, or rated, in which way they fit the selected policy criteria. This fit is indicated by '1' or'0' to denote levels of the various criteria, so that the matrix elaborated will indicate, e.g. for cost-effectiveness '1' when the intervention is CE and '0' if it is not. *To illustrate computation, in *Table 3*matrix, Atrial fibrillation intervention is rated as '0' for Disease severity criteria that is "no severe", '1' as providing significant individual benefit, '0' as not indicated for middle age group, '1' as indicated for high age group, '1' as the intervention is cost effective... when the mapping for all criteria is completed, the CI is calculated, for all interventions, based on the main effect additive utility model (formula *1), so that Atrial fibrillation will have as CI: 0*1.08 + 1*1.2638+0* - 0.2004+1*-1.117+1*1.5045+0*0.3714+1*0.3566 = 2.0079. Asthma no control will have as CI:1*1.08+1*1.2638-1*0.2004-1*1.117+1*1.5045+1*0.3714+1*0.3566 = 3.2589. The selection probability for each interventions is calculated, based on the general equation: P = EXP (CI)/1+ EXP (CI), the exponential (EXP) function returns e raised to the nth power, where e = 2.71828183. The selection probability for Atrial fibrillation is computed as:

$${\text{ε}}^{2.0079}/1+{\text{ε}}^{2.0079}\;\text{or}\;2.71^{2.0079}/1+(2.71^{2.0079})=7.4476/8.4476=0.88.$$

**Table 3:** Composite League Table (COPD: Chronic obstructive pulmonary disease, K/cancer)

		Norway Coefficients	1,08	1,2638	-0,2004	-1,117	1,5045	0,3714	0,3566			
**Intent N°**	**CLINICAL**	**Intervention**	**CRITERIA**							**Comp**	***Select***	***Rank***

	**CONDITION**									**Index**	***Prob***	

			**Dis Sev**	**Ind Ben**	**Age Mid**	**Age High**	**CER**	**Tot benef**	**WTSub**			

CVD1	Angina pectoris	as per Guidelines[apG] : invest, treat (med-stent surg)	1	1	0	1	1	1	1	3,4593	0,9695 07279	1

CVD2	Atrial fibrilation	diag, trat anticoag,	0	1	0	1	1	0	1	2,0079	0,8816 24035	13

CVD3	Heart failure	Diagn, eval, Med treat	1	1	0	1	1	1	1	3,4593	0,9695 07279	1

CVD4	High Cholesterol	Preventive screening /statin treatment	1	0	1	1	1	1	1	1,9951	0,0880 28164 9	14

CVD5	Hypertension	Screening, lifestyle (exerc, diet),ACE treat	1	0	1	1	1	1	1	1,9951	0,0880 28164 9	14

Resp 6	COPD Stade 1-2	spiro diag, X-ray, gaz anal, treat adapt, rehab	1	1	1	1	1	1	1	2,1789	0,8983 38657	8

Resp 7	COPD Stade 3-4	spiro diag, X-ray, gaz anal, Br dilat, rehab- O2-Hospi	1	1	1	1	1	1	1	3,2589	0,9629 91608	3

Resp 8	Asthma no control	diag, stress test..	1	1	1	1	1	1	1	3,2589	0,9629 91608	3

Resp9	Asthma control	treat adjust: inhal cortic, Beta agonist- leukot inhibit	1	1	1	1	1	1	1	3,2589	0,9629 91608	3

Resp 10	Tabacco Use	Prevention (tax-advert ban...)	1	0	1	1	1	1	1	1,9951	0,8802 81649	14

Neo 11	Colon/rectum K	Surgery with/ without adjuvant treat	1	1	1	1	1	1	1	3,2589	0,9629 91608	3

Neo 12	Breast K	Surgery with adjuvant treat	1	1	1	1	1	1	1	3,2589	0,9629 91608	3

Neo 13	Lung K	Surgery with/without abjuvat treat	1	0	1	1	1	1	1	1,9951	0,8802 81649	14

Neo 14	colon polyps	Screening blood test/colonoscopy	0	1	1	1	1	1	1	2,1789	0,8983 38657	8

Psy 15	Unipolar depressive Disorder 1	Med treat outpatient setting/ Gen Pract	0	1	1	1	1	1	1	2,1789	0,8983 38657	8

Psy16	Unipolar depressive disorder 2	Med treat/ psycho In Hospital setting	0	1	1	1	0	1	1	0,6744	0,6624 87692	20

psy17	Alcohol use disorders	Tx on beverage/legal age/advert ban	0	0	1	1	1	1	1	0,9151	0,7140 42646	18

psy 18	Alzheimer & other dementials	comprehensive in-home care	0	0	1	1	1	1	1	0,9151	0,7140 42646	18

Psy 19	Alzheimer & other dementials	Nursing home/hospital care	0	0	1	1	0	1	1	- 0,5894	0,3567 72534	21

Sen org20	Hearing loss	hearing aid	0	1	1	1	1	1	1	2,1789	0,8983 38657	8

Sen org21	refractive errors	optical correction	0	1	1	1	1	1	1	2,1789	0,8983 38657	8

The research proposal was submitted for ethical approval to the IRS at the Johns Hopkins Bloomberg School of Public Health, and has been waived from requiring any formal ethics approval.

## Results

All 34 respondents completed and returned the whole survey questionnaire. Table 4 shows the results of the random effects regression model. A positive sign for a particular level implies a positive impact on the probability of choosing interventions with that level. As mentioned, the absolute values of the regression coefficients indicate their relative importance in priority setting, in that respect, cost-effectiveness, individual benefits and severity of diseases are the most important criteria. Except for middle age group and the willingness-to-subsidize criterion, all coefficients showed to be significant, and their signs had the expected direction.

**Table 4:** Results of the random effects regression

CRITERIA	Coefficient	Std. Err	P-value
Cost-effectiveness	1.5045	.14	0.000
Individual Benefits	1.2638	.14	0.000
Severity	1.080	.14	0.000
Age target group Mid	-.2004	.17	0.24
Age target group High	-1.1170	.18	0.003
Willingness to Subsidize	.3566	.1391	0.01
N° of Beneficiaries	.3714	.1394	0.008

Table 3 shows the composite league table results. The interventions with the highest priority are those addressing cardio-vascular diseases, and next the respiratory diseases. Those receiving the lowest attractively are related to mental health.

## Discussion

Norwegian policy makers consider cost effectiveness, individual benefits and severity of disease as important criteria for priority setting of interventions. The findings are in line with those that one *a priori *may expect for a high-income country with universal coverage where a willingness to subsidize is less relevant. While using generic criteria based on other settings, our study confirms that the policy makers are consistent and accurately weigh criteria that the Patients Rights Act prescribes. These are all criteria that patients need to meet to qualify for needed specialized health care and to receive the status of 'entitled patient'.

The results from composite league table can be considered indicative. It provides an insight in intervention priorities they are giving or neglecting to address chronic diseases. Only if cardiovascular diseases and respiratory diseases are properly addressed, mental health conditions - such as depression, alcohol disorders and Alzheimer's disease - may become attractive. This may be in conflict with already existing central government initiatives that aim to promote control of neuropsychiatric conditions, in particular dementia [20].

A limitation in our study is that we did not use well defined and easily quantifiable criteria based on locally formulated standards. Recognizing this, we do not avoid a realistic debate on policy setting in Norway as the criteria selected ex ante are close to the country specific and country-relevant, as well as the levels of attributes. Our present full set of criteria chosen describes the most important generic aspects of a health intervention as also is found in the two large reviews [6,7]. Also, our findings are comparable to the preferences of Dutch policy makers [9]. These two countries seem to favor efficiency to equity. Additionally, the trends in weights among Dutch health care groups are similar. Moreover, interventions will certainly have different perceived or measured characteristics in other countries. Some of criteria may not be relevant in other countries, yet this is accounted for by the country-specific weights that policy-makers in other settings may give to those criteria.

The second constraint i.e. the assumed independence of the criteria as they are defined in the DCE is important. Our approach is not only based on assumed independence but also assumed comprehensiveness and exclusiveness of the selected criteria, especially in the ranking of interventions. This is reflected and has been tested in the multi-variate regression approach, yet we cannot not exclude other relevant additional criteria in real-life settings. In addition, we also need to acknowledge that this type of MCDA provides a way to account for quantifiable elements of the decision-making process, yet other elements like feasibility, transaction costs, historical or local context, among others, may be difficult to incorporate into the approach [21]. The latter are much more related to implementation as to an informed and explicit priority setting. The present findings are important as they confirm current policies and do not lead to new insights in the approach of the policy makers. So, the study and the matching of criteria provides policymakers with an explicit structure to assess the extent to which current and future investments in health interventions in Norway may serve the country's societal objectives.

Health decision-makers make difficult decisions on the use of public funds either in a context-specific priority setting for reimbursement of interventions, prioritization between a few options within a constrained budget or, in a general policy setting where no specific choice has to be made in a specific resource allocation. At the moment, Norway is given only an average score in the OECD's new health system report [22]. A more formalized and explicit approach that includes methodological guidance, the use of health information and a transparent debate on policy formulation may improve the policy process [23]. Here, to balance policy makers taking ad-hoc or biased decisions, an approach like MCDA, while using a composite ranking method may be a valuable tool[2]. Ghana is a positive example and has successfully used explicit criteria to set its interventions priorities through the Strategic Plan for health 2007-2012 while other very divers countries with (almost) universal access systems continue to develop policy processes that includes both equity and efficiency factors such as in the UK, The Netherlands, Thailand, China, and others [15].

## Conclusions

Although this study used general priority criteria, three criteria showed to be relevant to Norwegian policymakers. The existing implementation of appropriate, transparent, and fair national guidelines is in line with the legal, and hence societal values and reflects the Patients Rights of Act. Priority rankings based on the same criteria show how MCDA can be used and may invite a discussion on priority policies across major disease areas. The findings allow for a prioritization based on existing features of the modern health care complexities that policy makers are facing.

## Competing interests

The authors declare that they have no competing interests.

## Authors' contributions

TD: Drafted the manuscript, carried out data extraction and analysis. FP: designed, conceived and coordinated the study, reviewed the draft. AM: contributed to the extraction, analysis and interpretation of the data, reviewed the draft. SY: contributed to the extraction, analysis and interpretation of the data. GB: contributed to the analysis and interpretation of the data. TH: Drafted the manuscript, collected the data, contributed to the analysis and interpretation of the data. LN: designed and conceived the study, contributed to the analysis and reviews. All authors read and approved the final manuscript.

## Pre-publication history

The pre-publication history for this paper can be accessed here:

http://www.biomedcentral.com/1472-6963/12/39/prepub
